# Neighbourhood-level deprivation indices and postpartum women’s health: results from the Community Child Health Network (CCHN) multi-site study

**DOI:** 10.1186/s12955-020-1275-x

**Published:** 2020-02-22

**Authors:** Vered Kaufman-Shriqui, Patricia O’Campo, Vachan Misir, Peter Schafer, Julia Morinis, Maxine Vance, Christine Dunkel Schetter, Tonse N. K. Raju, Marianne M. Hillemeier, Robin Lanzi, Vernon M. Chinchilli, for the Community Child Health Network (CCHN) of Eunice Kennedy Shriver National Institute of Child Health and Human Development, M. Vance, C. S. Minkovitz, P. O’Campo, P. Schafer, K. Walton, K. Wagenaar, M. Shalowitz, E. Adam, G. Duncan, A. Schoua-Glusberg, C. McKinney, T. McDade, C. Simon, E. Clark-Kauffman, L. Jones, C. Hobel, C. Dunkel Schetter, M. C. Lu, B. Chung, F. Jones, D. Serafin, D. Young, S. Evans, J. Ruffin, R. Woolard, J. Thorp, J. DeClerque, C. Dolbier, C. Lorenz, L. S. Sahadeo, K. Salisbury, L. Patchen, S. L. Ramey, R. G. Lanzi, N. Timraz, R. German, V. M. Chinchilli, R. Belue, G. Brown Faulkner, M. Hillemeier, I. Paul, M. L. Shaffer, G. Snyder, E. Lehman, C. Stetter, J. Schmidt, K. Cerullo, S. Whisler, J. Fisher, J. Boyer, M. Payton, V. J. Evans, T. N. K. Raju, L. Weglicki, M. Spittel, M. Willinger, Y. Bryan, M. Phillippe, E. Fuentes-Afflick

**Affiliations:** 1 Department of Nutrition Sciences, School of Health Sciences, Ariel University, Ariel, Israel; 2 The Center for Urban Health Solutions (C-UHS), St, Michael’s Hospital, Toronto, Canada; 3 Alma and Baxter Richard Chair in Inner City Health, St. Michael’s Hospital, 30 Bond St, Toronto, ON M5B 1W8 Canada; 4 Dalla Lana School of Public Health, University of Toronto, Toronto, Ontario, Canada; 5 Johns Hopkins Bloomberg School of Public Health, Baltimore, Maryland USA; 6 Baltimore Healthy Start, Inc 2521 N. Charles Street, Baltimore, MD 21218 USA; 7 Department of Paediatric Medicine, Hospital for Sick Children, Toronto, Ontario Canada; 8 Senior Director of Clinical Affairs and Quality Assurance, Baltimore Healthy Start, Inc, 2521 N. Charles Street, Baltimore, MD 21218 USA; 9 Department of Psychology, University of California, Los Angeles, CA USA; 10 Eunice Kennedy Shriver National Institute of Child Health and Human Development National Institutes of Health, Bethesda, MD USA; 11 Department of Health Policy and Administration, Pennsylvania State University, 504S Ford, University Park, PA 16802 USA; 12 Department of Health Behavior, School of Public Health, University of Alabama at Birmingham, 1665 University Blvd., 227 RPHB, Birmingham, AL 35294 USA; 13 Department of Public Health Sciences, A210, Penn State College of Medicine, 90 Hope Frive, Suite 2200, Hershey, PA 17033-0855 USA

**Keywords:** Maternal health, Postpartum period, Socioeconomic factors, Residence characteristics

## Abstract

**Background:**

Area-level socioeconomic characteristics have been shown to be related to health status and mortality however, little is known about the association between residential community characteristics in relation to postpartum women’s health.

**Methods:**

Data from the longitudinal, multi-site Community Child Health Network (CCHN) study were used. Postpartum women (*n* = 2510), aged 18–40 were recruited from 2008 to 2012 within a month of delivery. Socioeconomic data was used to create deprivation indices. Census data were analysed using principal components analysis (PCA) and logistic regression to assess the association between deprivation indices (DIs) and various health indicators.

**Results:**

PCA resulted in two unique DIs that accounted for 67.5% of the total variance of the combined all-site area deprivation. The first DI was comprised of variables representing a high percentage of Hispanic or Latina, foreign-born individuals, dense households (more than one person per room of residence), with less than a high-school education, and who spent more than 30% of their income on housing costs. The second DI was comprised of a high percentage of African-Americans, single mothers, and high levels of unemployment. In a multivariate logistic regression model, using the quartiles of each DI, women who reside in the geographic area of Q4-Q2 of the second DI, were almost twice as likely to have more than three adverse health conditions compared to those who resided in the least deprived areas. (Q2vs.Q1:OR = 2.09,*P* = 0.001,Q3vs.Q1:OR = 1.89,*P* = 0.006,Q4vs.Q1:OR = 1.95,*P* = 0.004 respectively).

**Conclusions:**

Our results support the utility of examining deprivation indices as predictors of maternal postpartum health.

## Introduction

Inequalities in residential neighbourhood socioeconomic deprivation have been shown to lead to disparities in the risk of premature mortality [1] and all-cause mortality [2]. Neighbourhood deprivation, distinct from individual socioeconomic status (SES), is independently associated with a wide range of adverse health outcomes, such as diabetes, [3, 4] cancer, [5] and chronic heart diseases [6, 7]. Neighbourhood factors may influence health by shaping health risk behaviours in pregnancy, sexual practices, and active, healthy living [8–10]. Living in deprived neighbourhoods is also negatively associated with perinatal health outcomes, such as low birth weight and preterm birth, which have life-course health effects [11–14]. Furthermore, neighbourhood deprivation is associated with adverse maternal health during pregnancy, including inadequate weight gain and pregnancy-induced hypertension [11]. It is not known, however, whether neighbourhood contextual features impact maternal postpartum health.

The postpartum period poses physical and emotional challenges for women; nearly 70% of women report at least one physical health problem within the first 12 months postpartum [15]. Adverse health outcomes during this period can affect women’s ability to function and care for their newborn, as well as influencing future fertility and productivity [16]. Most research on postpartum health has examined maternal mental health and/or chronic disease and focused on individual-level health behaviours (e.g., smoking) and general SES factors such as poverty and maternal low education [17]. Neighbourhood deprivation may be of greater concern for women during the postpartum period in Western societies, especially for women who live alone, with little or no help for domestic work and childcare. If they also lack easy access to adequate community and physical resources and services, the burden of deprivation can be worse, preventing women from maintaining a healthy lifestyle [18].

Neighbourhood socioeconomic indicators are widely used in maternal and child health (MCH) research to assess neighbourhood characteristics and adversity [18, 19]. However, a wide variety of variables has been used [20]. The most common measures reported in MCH research [13, 18, 19] include income/poverty [21] employment, [21–23] family composition, [20] and area racial composition [24]. Findings, from various sites in the USA show that women living in neighbourhoods with high unemployment, low education, poor housing and high poverty had increased odds of preterm birth, low birth weight and small for gestational age [21, 23, 24]. Less commonly reported variables include housing quality and crowding, [23] education, [25] occupation, [26] and immigration [27]. Suggested explanations for the role that thouse variables have is associasion with health emphasize the presence of resources, amenities and infrastructure to accommodate the interests and activities of for instance, more highly educated groups (e.g., the presence of high quality schools, recreation facilities, and access to grocery stores) [21]. Previous studies have examined both individual-level socioeconomic factors [13] and composite [28] or generated indices [13, 21, 23]. While indices allow the shared and total variance of correlated socioeconomic factors to be accounted for, individual factors allows for the identification of each indicator’s unique contributions, and miss the more complex and often, less intuitive associations to additional socioeconomic factors. Since there is limited research on the contextual determinants of MCH, it is necessary to broadly explore neighbourhood characteristics based on theoretical explanations [12, 21]. Traditional approaches to MCH disparity have not included the knowledge and perspective of community residents most affected by the research and do not appear to be theoretically associated with the research outcomes.

In this paper, we have used data from a multi-site study, the Community Child Health Network (CCHN) study, to develop specific neighbourhood deprivation indices (represented by principal components). We hypothesized that higher neighborhood adversity would be associated with higher prevalence of adverse health conditions.

## Methods

### Study population

The CCHN is a collaborative partnership of five university departments and community partners. The following study sites were included in our sample: Washington, D.C.; Baltimore, Maryland; Los Angeles County, California; Lake County, Illinois; and seven counties in eastern North Carolina (Pitt, Greene, Washington, Tyrell, Martin, Bertie, and Edgecombe). CCHN developed a Preconception Stress and Resiliency Pathways (PSRP) model by building local and multi-site community-academic participatory partnerships that reviewed relevant findings diverse disciplinary and community perspectives; and identified the major themes of stress and resilience among women within the context of families and communities [29]. The original sample size calculation of the CCHN study is described elsewhere [30]. Participants were recruited using a population-based sampling method. Inclusion criteria were maternal age 18–40 years; self-identification as either “Black or African American”, “Hispanic or Latina”, or “White”; residence in the study catchment areas; and birth of an infant at ≥20 weeks gestation. Socioeconomically disadvantaged mothers and those delivering preterm infants were oversampled. Exclusion criteria were the inability to understand English or Spanish or to provide informed consent, child birth order of 4th or higher, residence in the study area < 6 months, incarceration or other circumstances preventing study participation, or plans for surgical sterilization after birth. Ethics approval was sought from and given by the respective Research Ethics Boards at the institutions affiliated with each of the PIs in the five study sites, a written informed consent was obtained for the participants, which included permission to access medical records of the mother and newborn.

### Neighbourhood deprivation

The process by which the CCHN Community Committee members (community PIs and Co-Investigators) selected variables was informed by indicators from previous research [12, 19, 30] and the community experience of the Committee members. First, the Committee developed an initial listing of broad categories of interest; the CCHN Data Coordination and Analysis Centre (DCAC) then provided the corresponding descriptions of 2006–2010 American Community Survey (ACS) variables and each ACS variable was reviewed to determine whether it captured a community-level factor that could plausibly be associated with stress and related health outcomes.

Variables recommended as high priority by the CCHN Community Committee were derived from the census data to develop the DIs. Eligible participants’ home addresses were geo-referenced, assigning the address to the block (street segment) level. Seven socioeconomic and demographic domains were chosen, including poverty, housing, employment, education, immigration, area racial composition and sex composition (men-to-women ratio). In total, 14 census variables were created to represent the seven domains and analysed using principal component analysis (PCA).

### Health outcomes

We examined six health outcomes that were chosen by the CCHN being components of maternal allostatic load (a composite biomarker index of cumulative stress that can lead to disease outcomes and health disparities), [29, 31] and tested those measures in relation to neighbourhood deprivation. (1) Body mass index (BMI): Weight in pounds and ounces and height in inches were measured using standardized equipment and procedures and compared to a National Heart, Lung and Blood Institute (NHLBI) chart to determine BMI. (2) Waist-to-hip ratio (WHR): Waist and hip circumferences were measured while standing and recorded to the nearest centimetre, and the ratio of the two values was calculated. (3) Systolic and (4) diastolic blood pressure: Blood pressure readings were recorded while participants were seated using standardized digital sphygmomanometers (5) Glycosylated haemoglobin and (6) HDL cholesterol: Blood was collected and analysed for glycosylated haemoglobin (A1c) (%) and HDL cholesterol (mg/dL). All assays were performed on blood spots by a commercial Cleo approved reference laboratory (ZRT) Laboratory, Beaverton, OR (www.zrtlab.com), except in the early months of the study when cholesterol was analysed in the field using Cholestech LDX. A composite score was computed using a cumulative count, ranging from 0 to 6, indicating at or above the following clinical cutoff values. BMI ≥30, WHR ≥0.8, [31, 32] A1c ≥5.6, [33] average systolic blood pressure (SBP) ≥135, average diastolic blood pressure (DBP) ≥85, [34, 35] and HDL cholesterol ≤40 [36, 37]. This composite score was then dichotomized to create a higher risk group (composite scores of 3–6) and a lower risk group (scores of 0–2).

### Stress outcomes

Two stress outcome variables were selected for analysis based on the Community Committee’s recommendations and tested for their association with the DIs. Financial stress was calculated as a composite score based on the sum of five questions, providing a score ranging from 0 to 5, with 5 being the greatest financial strain. Perceived stress was a composite score (range 0–40) based on ten questions, with 40 being the greatest perceived stress.

### Statistical analyses

Statistical analyses were performed using SAS version 9.3. Dimension reduction was conducted using PCA, a method frequently used in neighbourhood-level research to create sociodemographic indices for inclusion in statistical models [18]. All 14 census variables which represented the seven socioeconomic domains were analysed using PCA procedure. PCA captures the total area-level variance explained by the selected variables, with the factor loadings representing the correlation between the variable and the factor. Variables were included based on the a priori condition that correlations be greater than 0.5. We calculated quartile cut points (Q1–Q4) from the continuous DI measures for all CTs, with Q1 being the least deprived area in the CT and Q4 being the most deprived. Associations between the selected health outcomes and the principal component (PC) loadings were examined using logistic regression models. For the continuous stress outcomes, a generalized linear model (GLM) was fitted. Each model’s goodness-of-fit was checked by the Hosmer–Lemeshow test, and the model with the best fit was chosen. Confidence intervals (95% CI) and *P*-values < 0.05 were considered statistically significant.

## Results

**Fig. 1:**
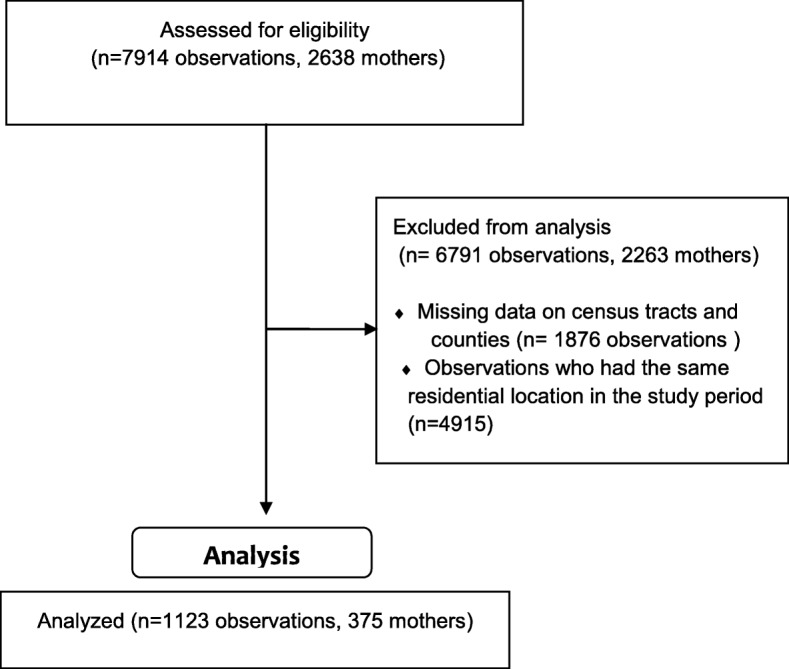
Flow chart of the study data

The CCHN study consisted of data collection on 2638 mothers measured at three time points (7914 observations). After removing observations missing data on census tracts and counties (1876) and mothers who had the same residential location throughout the study (4915), our final sample included 1123 observations uniquely identified, for these, DIs were created at the CT and county level (Fig. 1).

Socioeconomic characteristics of the study sites are presented in Table 1. CTs had population counts ranging from a mean of 4823 in NC to 3532 in Baltimore.

**Table 1:** Sociodemographic descriptions of each Community and Child Health Network (CCHN) study area. Means, counts or proportions (standard deviation), population, and top two quartiles (50%) of the population in the Census Tract (CT) by area, and deprivation index (DI) quartiles 1 and 2, year 2006 USA, census data

	Baltimore	Lake County, Illinois	DC	LA	NC
Number of CTs	260	142	249	381	91
Number of CTs, top 2 quartiles of factor 1	98	52	96	288	21
Number of CTs, top 2 quartiles of factor 2	169	20	186	142	45
Sociodemographic Characteristics – Mean (SD)					
Tract Population (complete sample in the CT)	3532 (1502)	4778 (1543)	3813 (1387)	4192 (1273)	4823 (1706)
Tract population, top 2 quartiles of factor 1	3058 (1319)	4641 (1497)	3975 (1388)	4245 (1270)	4550 (1681)
Tract population, top 2 quartiles of factor 2	3430 (1485)	3942 (1729)	3581 (1267)	4423 (1373)	4782 (1666)
% poor families (under 1.30 of the poverty line) w/ children < 18 years	15.6 (13.6)	9.3 (10.1)	13.5 (12.7)	20.5 (15.7)	17.1 (11.5)
Top 2 quartiles of factor 1	24.3 (14.4)	17.9 (10.6)	17.9 (13.1)	25.3 (14.6)	25.7 (15.5)
Top 2 quartiles of factor 2	20.4 (13.9)	24.5 (12.1)	16.1 (13.3)	29.2 (13.5)	23.5 (12.1)
% housing occupancy > 1.0 (combining owner and renter)	2.1 (2.4)	3.1 (3.7)	3.7 (3.8)	15.7 (13.3)	2.0 (2.1)
Top 2 quartiles of factor 1	3.34 (2.9)	6.6 (3.8)	6.34 (4.5)	19.9 (12.2)	3.5 (2.9)
Top 2 quartiles of factor 2	2.5 (2.6)	6.7 (3.9)	3.9 (3.9)	19.5 (11.6)	2.2 (2.3)
% housing units (combining owner and renter) lacking complete plumbing	0.6 (1.2)	0.5 (0.9)	0.4 (1.3)	0.7 (1.5)	0.7 (1.3)
Top 2 quartiles of factor 1	0.49 (1.1)	0.71 (1.3)	0.23 (0.6)	0.87 (1.7)	1.2 (1.6)
Top 2 quartiles of factor 2	0.61 (1.3)	0.80 (1.4)	0.41 (1.32)	0.72 (1.2)	0.9 (1.5)
% households with housing costs > 30% of income	35.2 (10.5)	38.5 (9.7)	39.7 (12.6)	52.6 (16.7)	30.6 (9.7)
Top 2 quartiles of factor 1	38.8 (11.6)	45.9 (10.1)	44.1 (14.7)	56.1 (16.6)	37.8 (11.9)
Top 2 quartiles of factor 2	37.2 (9.9)	48.2 (9.9)	41.3 (12.9)	56.9 (15.5)	34.5 (10.3)
% foreign born in the CT	8.1 (7.6)	17.8 (11.9)	16.6 (15.4)	35.2 (14.3)	4.5 (4.9)
Top 2 quartiles of factor 1	8.3 (9.6)	28.7 (11.3)	25.8 (18.2)	39.7 (11.9)	5.9 (6.5)
Top 2 quartiles of factor 2	6.0 (6.3)	23.7 (14.10)	13.1 (13.9)	34.3 (12.9)	4.1 (5.8)
Sociodemographic Characteristics	Baltimore Mean (SD)	Lake County, Illinois Mean (SD)	DC Mean (SD)	LA Mean (SD)	NC Mean (SD)
% children of single-parent female householder (own children < 18 years)	21.3 (13.8)	10.3 (8.2)	22.1 (14.1)	16.1 (9.7)	15.8 (9.4)
Top 2 quartiles of factor 1	28.1 (13.3)	14.68 (8.8)	23.84 (14.3)	18.45 (9.3)	19.95 (13.7)
Top 2 quartiles of factor 2	27.7 (12.5)	23.3 (11.2)	26.3 (13.5)	24.4 (7.1)	20.3 (10.5)
% females > 25 < HS education in the CT	19.3 (11.5)	14.0 (13.1)	16.1 (10.7)	28.8 (21.0)	17.4 (8.9)
Top 2 quartiles of factor 1	29.7 (10.1)	27.7 (12.3)	24.76 (10.2)	36.05 (18.5)	25.65 (6.3)
Top 2 quartiles of factor 2	22.3 (11.3)	29.9 (13.3)	17.06 (10.4)	36.39 (16.1)	21.43 (7.5)
% males > =25 < HS education in the CT	22.0 (13.2)	14.4 (14.1)	17.7 (11.9)	29.1 (21.4)	21.2 (11.2)
Top 2 quartiles of factor 1	33.4 (11.5)	28.8 (13.8)	27.3 (11.5)	36.7 (18.6)	33.6 (8. 9)
Top 2 quartiles of factor 2	25.9 (13.0)	32.0 (15.2)	18.7 (11.2)	39.2 (17.3)	26.3 (10.6)
% female 22–44 unemployed in the CT	7.2 (6.6)	5.7 (5.1)	8.8 (7.4)	6.6 (5.0)	9.2 (7.3)
Top 2 quartiles of factor 1	8.8 (7.4)	8.3 (5.76)	9.4 (7.7)	7.1 (5.2)	13.2 (10.4)
Top 2 quartiles of factor 2	9.2 (7.0)	13.4 (6.23)	10.6 (7.6)	8.9 (5.8)	12.9 (8.1)
% male 22–44 unemployed in the CT	9.3 (9.6)	6.4 (5.0)	9.2 (7.9)	7.2 (5.0)	7.9 (6.4)
Top 2 quartiles of factor 1	12.1 (11.2)	7.0 (5.2)	9.3 (6.7)	7.6 (5.2)	10.3 (6.9)
Top 2 quartiles of factor 2	12.3 (10.3)	9.6 (5.8)	10.9 (8.2)	9.9 (5.3)	10.73 (7.1)
% renter-occupied units with gross rent > 30% of income in the CT	57.0 (14.2)	53.3 (17.6)	53.7 (13.2)	60.1 (12.7)	61.0 (13.5)
Top 2 quartiles of factor 1	61.3 (12.4)	54.8 (13.2)	54.8 (11.4)	62.8 (10.7)	65.2 (14.5)
Top 2 quartiles of factor 2	61.2 (11.8)	59.4 (13.7)	54.8 (12.4)	65.9 (9.8)	63.5 (13.7)
% black or African American in the CT	57.8 (35.3)	10.2 (17.7)	65.9 (29.6)	18.6 (21.4)	41.5 (21.9)
Top 2 quartiles of factor 1	66.7 (32.8)	54.8 (13.2)	54.8 (11.4)	62.8 (10.7)	65.2 (14.5)
Top 2 quartiles of factor 2	78.7 (21.9)	40.2 (29.1)	78.9 (19.1)	36.0 (22.9)	56.4 (20.0)
% Hispanic in the CT	3.9 (6.3)	20.8 (21.5)	12.0 (14.6)	45.6 (29.9)	5.3 (6.3)
Top 2 quartiles of factor 1	5.9 (9.1)	43.07 (20.4)	22.52 (18.3)	56.4 (25.6)	9.19 (9.7)
Top 2 quartiles of factor 2	2.9 (5.5)	38.43 (22.9)	9.72 (13.4)	54.2 (23.7)	5.09 (6.7)
% males 18–44 in the CT	48.7 (14.9)	49.3 (9.5)	48.1 (14.6)	49.8 (12.1)	49.6 (12.0)
Top 2 quartiles of factor 1	50.3 (16.1)	50.7 (11.6)	50.3 (13.1)	49.7 (11.8)	49.2 (15.3)
Top 2 quartiles of factor 2	47.8 (15.0)	48.2 (17.6)	46.9 (14.7)	46.9 (11.9)	47.8 (11.8)

The CCHN is a collaborative partnership of five university departments and community partners. The following study sites were included in our sample: Washington, D.C.; Baltimore, Maryland; Los Angeles County, California; Lake County, Illinois; and seven counties in eastern North Carolina

Significant variability was observed for the derived sociodemographic census variables. On average, Los Angeles (LA) County, California, appeared to be the most economically deprived, with 20.5% of families with children under 18 lived at ≤130% of the federal poverty line and 52.6% of households had housing costs exceeding 30% of household income. The CTs with the highest percentages of foreign-born residents (35%) were also in LA County. Race and ethnic composition varied across sites; for example, 65.9% of the population in Washington, D.C. CTs was black and African American, whereas 45.6% of the population in LA County CTs was Hispanic or Latina.

### Generation of indices

**Fig. 2:**
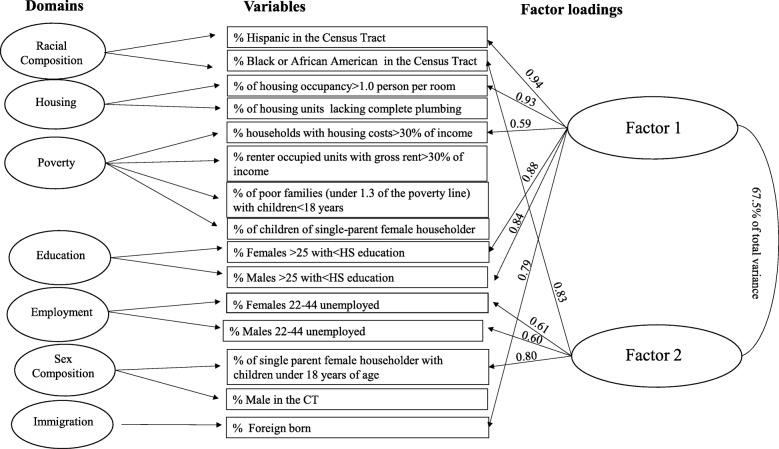
Principal component indices generation model: Area-level socioeconomic and residential characteristics in the Community and Child Health Network (CCHN) study*. * Values represent the factor loads

Two poverty-related variables loaded equally in the first stage of the DIs generation:(1) the percentage of households with children under 18 living in poverty, and (2) the percentage of renter-occupied units with gross rent higher than 50% of income in the CT. In order to produce unique DIs, these variables were not included in the second stage of the PCA. Two additional variables (% of males 18–44, % of housing units lacking complete plumbing) were not entered in the second stage, since they loaded lower than 0.2 on the first two DIs. PCA performed on the remaining variables generated two final indices capturing unique characteristics of the study population. Factor loadings are presented in Tables 2 and 3. The two indices accounted for 67.5% of the total variance, the first index accounted for 43.3% of the total variance, and the second index added 24.2%. (Fig. 2) A third component that added 8.0% to the explained variance was not retained.

**Table 2:** Community Child Health Network (CCHN) loadings for the first deprivation index

Variables	Principal component loading
% Hispanic or Latina in the Census Tract	0.94
housing occupancy > 1.0 person per room (combining owner and renter)	0.93
% females ≥25 with <HS education in the Census Tract	0.88
% males ≥25 with <HS education in the Census Tract	0.84
% foreign born in the Census Tract	0.79
% households with housing costs > 30% of income (aggregate across income categories and owner-occupied) in the Census Tract	0.59

**Table 3:** Community Child Health Network (CCHN) loadings for the second deprivation index

Variables	Principal component loading
% black or African American in the Census Tract	0.83
% single-parent female householder with children < 18 years of age in the Census Tract	0.80
% female age 22–44 unemployed in the Census Tract	0.61
% male age 22–44 unemployed in the Census Tract	0.60

The first DI correlated highly with areas characterized by high percentages of Hispanic and foreign-born residents with less than a high-school education (men and women ≥25 years), living in crowded housing, and spending more than 30% of their income on housing. The second DI correlated highly with areas characterized by high percentages of Black residents, single mothers, and parental unemployment.

Significant differences in socioeconomic heterogeneity in the all-site DI by each of the five study areas. DIs ranged from − 1.63 to 3.91, with the DI for the smallest site, North Carolina (91 CTs), having an average factor loading of − 0.59, and largest site, LA County (381 CTs), having an average loading of 0.82. The second DI had an average score of − 0.71 in Lake County, compared to 0.56 in Washington, D.C. These values indicate that the DIs were consistent across study areas, despite significant geographic and sociodemographic variability.

The deprivation indexes were examined for their association with maternal financial stress and perceived stress. Women in the third quartile of DI2 were at twice the risk of reporting life-long financial stress compared to women in the lowest quartile (OR = 2.08, 95% CI = 1.34–3.22, *p* = 0.001). Women in the second quartile of DI2 had 1.6 times the risk of reporting life-long financial stress compared to women in the lowest quartile (OR = 1.61, 95% CI = 1.03–2.56, *p* = 0.04). Women in the third quartile of DI1 had significantly less perceived stress than women in the most deprived quartile (Est = − 1.26, *p* = 0.04). Women in the second quartile of DI 2 had significantly more perceived stress than women in the most deprived quartile (Est = 1.25, *p* = 0.04; Est = 2.16, *p* = 0.003; Est = 1.39, *p* = 0.02 respectively).

### Multivariate analysis

Crude odds ratios comparing Quartiles 2–4 with Quartile 1 for each DI for selected metabolic risk indicators were calculated. Women represented by DI1 were at higher risk for central obesity, with the most deprived group (Q4) at almost double the risk compared to the least deprived group (OR = 1.91, 95% CI:1.24–2.94, *p* = 0.003). Among the same population and across all area-level categories, a higher risk for HDL cholesterol (≤40), was found (OR = 1.74, 95% CI:1.18–2.54, *p* = 0.004).Area-level deprivation was inversely associated with the risk of clinically significant high systolic and diastolic blood pressure with the most deprived group (Q4) at third of the risk for clinically significant high systolic blood pressure and about half of the risk to clinically significant high diastolic blood pressure compared to the least deprived group (OR = 0.34, 95% CI:0.16–0.74, *p* = 0.006; OR = 0.46, 95% CI:0.27–0.80, p = 0.006 respectively).

Among women represented by DI2, models were significant for BMI ≥30 and A1c ≥5.6%; DI2 was associated with a higher risk of obesity among all area-deprived populations compared to the least area-deprived group (Q4-Q2 vs. Q1) with the most deprived group (Q4) at almost triple risk for obesity compared to the least deprived group (OR = 2.56, 95% CI:1.66–3.95, *p* < 0.001). The risk of having A1c levels ≥5.6% was higher among women in Q4-Q2 than Q1, with the most deprived group (Q4) at almost double the risk compared to the least deprived group (OR = 1.93, 95% CI:1.28–2.90, *p* = 0.001).

The overall model of the composite risk score was significant among the population represented by DI2; the risk of belonging to the higher risk group (composite score 3–6) was higher among the DI2 Q2–42 population than the least area-deprived group (Q1), p for trend< 0.001. Women in Q4, Q3, and Q2 were at a significantly higher risk than those in the lowest quartile (95% CI: 0.12–0.74, *p* = 0.007; 0.06–0.65; *p* = 0.02; and 0.25–0.79, *p* < 0.001 respectively). In comparison with LA, two sites were at significantly higher health risk: Baltimore (95% CI: 0.27–0.88, *p* = 0.0002) and Washington, D.C. (95% CI: 0.06–0.65, *p* = 0.02). There was no significant correlation between DI1 and the higher risk group.

## Discussion

In this study, we created neighbourhood DIs that capture cross-disciplinary and community perspectives on area-level deprivation. We examined the relationship between neighbourhood deprivation and maternal postpartum health across five geographic areas in the United States. DIs were comprised of a set of neighbourhood characteristics identified as being highly relevant to women’s health. We found a significant association between neighbourhood deprivation and health outcomes.

Most research on area deprivation and women’s health has focused on adverse neonatal outcomes and premature birth or delivery complications [12, 13, 36, 38, 39]. The association between area-level deprivation and adverse health outcomes has been documented only among middle-aged and older women in the general French population, notably BMI, central obesity and the metabolic syndrome [40]. In that study, the prevalence of diabetes increased with deprivation and was more than two times higher among women in deprived areas than non-deprived women. Our results add to the literature on younger North American women’s health in the postpartum period.

Postpartum period may cause major changes in women’s social lives,physical and mental health; nearly 70% of women report at least one physical health problem within the first 12 months postpartum [15]. The problem is reported to be of moderate severity for 25% of women and severe for 20%. Pregnancy-related health outcomes have a significant impact on women’s abilities to work, look after their children, and undertake household chores, as well as their overall mental health.

Evidence suggests that the availability of energy-dense, nutrient low-food (e.g., fast food) is associated with neighbourhood deprivation [16, 41]. Thus, individual dietary habits are influenced by such neighbourhood factors as food affordability, availability, and accessibility [10, 17].

Among middle-aged and older women, area-level deprivation is associated with lower consumption of fruit and vegetables, less physical activity, and more smoking behaviours [19]. Mothers of newborns may be even more affected by the lack of food accessibility and affordability, since they may need to spend more to take care of their infant and may not have the flexibility to seek high-quality food.

Neighbourhood deprivation may also contribute to maternal adverse health outcomes through its impact on physical activity. A highly walkable neighbourhood promotes healthy habits, reducing the risk of obesity and type 2 diabetes mellitus [42]. A systematic review reported that more people tend to engage in physical activity in neighbourhoods equipped with easily accessible, appealing facilities such as recreational parks, sport clubs, and clean sidewalks [43].

We found significant differences in the relationship between neighbourhood deprivation and health outcomes among women of different races and ethnicities. Area-level deprivation was inversely associated with the risk of clinically significant high systolic blood pressure among the population represented by DI1. A lower prevalence of high blood pressure among Hispanic adults than other ethnic groups has been documented despite a higher risk of central obesity and other risk factors. According to 2008 estimates, 18% of Hispanic adults 18 years of age or older have been diagnosed with hypertension, compared to 27% of non-Hispanic Whites and 32% of non-Hispanic Blacks [44]. In our sample, racial variation may stem from high levels of segregation, since people of different races/ethnicities frequently live in different neighbourhoods [45]. An increased risk of diabetes was found in CTs with high percentages of black women, but not in those with high percentages of Hispanic women despite the presence of central obesity, which is a major contributor to diabetes risk. In a meta-analysis, higher HA1c levels were observed among African Americans than non-Hispanics and whites [46]. Ethno-racial differences in the risk of high blood pressure and diabetes should be further investigated at the individual level. The burden of metabolic risk factors among postpartum women underscores the urgent need to understand socioeconomic risk factors, in order to recommend targets for intervention.

These findings should be interpreted in light of the study’s limitations. First, our sample was selected in specific U.S.A. locations, with oversampling of low socioeconomic and minority women; the results may not be generalizable to the entire population. The two unique DIs were generated in a result of principle components analysis which used a specific are-level characteristics and cannot be directly used but rather suggest a methodology to those investigating the influence of the socio-economic context on health. Second, our data included women with type 2 diabetes mellitus prior to pregnancy and gestational diabetes mellitus during pregnancy. To assess the impact of this factor, we repeated the analyses with these women excluded and our results remained the same. Third, our results relate to the effect of the socio-economic context on health and thus enable a multilevel research, which will integrate individual-level data. Fourth, our data do not include information regarding access to services and infrastructures that might be important in the postpartum period, such as food stores, well-baby care clinics, and parks.

## Conclusions

In conclusion, our results are consistent with past research and demonstrate that indicators of neighbourhood deprivation based on a broad set of area-level characteristics are useful for understanding metabolic risk in different racial groups and a wide variety of geographic settings. Furthermore, while variation may be observed across geographic areas, this effect appears to be similar across diverse settings. In future research, investigators should use our derived index to determine whether it is useful to predict other adverse health outcomes. Due to the cross-sectional nature of the study, it is hard to draw direct implications to the clinical setting; however, our findings suggest that two specific residential characteristics pose a greater risk on postpartum women to develop chronic conditions. If a clinic serves women at such an area, physicians working in it should be aware of the higher risk to develop chronic conditions among and screen for it.

Researchers investigating the pathways connecting neighbourhood environment to maternal postpartum metabolic risk should also include individual-level data, such as family history and individual lifestyle.

## Data Availability

Datasets and research resources associated to this study can be accessed via NIH.com, at: https://www.nichd.nih.gov/research/supported/cchn. CCHN data are available in NICHD’s Data and Specimen Hub (DASH). https://dash.nichd.nih.gov/

## Abbreviations

ACS: American Community Survey
CBPR: community-based participatory research
CCHN: Community Child Health Network
CT: Census Tract
DCAC: Data Coordination and Analysis Centre
DI: Deprivation Index
GEE: Generalized Estimating Equation
PCA: Principal Component Analysis
